# Chemogenetic Perturbation of the Posterior But Not Anterior Cerebellum Reduces Voluntary Ethanol Consumption

**DOI:** 10.1523/ENEURO.0037-23.2023

**Published:** 2023-09-18

**Authors:** Paula A. Zamudio, Dominic Gioia, Christina Glaser, John J. Woodward

**Affiliations:** Department of Neuroscience, Medical University of South Carolina, Charleston, SC 29425

**Keywords:** alcohol dependence, alcohol drinking

## Abstract

The cerebellum communicates with brain areas critically involved in control of goal-directed behaviors including the prefrontal and orbitofrontal cortices and midbrain and basal ganglia structures. In particular, the posterior cerebellum is important for cognitive flexibility and has been implicated in alcohol and drug-related memory. We hypothesized that the cerebellum, through its multiple connections to reward-related brain circuitry, regulates alcohol consumption. To test this, we expressed inhibitory designer receptors exclusively activated by designer drugs (DREADDs) in molecular layer interneurons (MLIs) in anterior (IV–V) or posterior (VI–VIII) cerebellar lobules of male and female mice and activated them during alcohol drinking sessions. In a home-cage drinking paradigm, alcohol consumption was significantly decreased by clozapine-N-oxide (CNO) or deschloroclozapine (DCZ) administration in male mice expressing DREADDs in posterior but not anterior lobules. CNO/DCZ injections did not affect drinking in DREADD expressing female mice or in male mice expressing the control vector. Activation of DREADDs expressed in anterior or posterior lobules had no effect on sucrose or quinine consumption in male or female mice. During operant self-administration sessions, DCZ decreased the number of licks and bouts in male but not female mice expressing DREADDs in posterior lobules with no effect in control vector mice. Performance on an accelerated rotarod was unaffected by chemogenetic manipulation while distance traveled in the open field was decreased by DREADD activation in anterior but not posterior lobules. These results indicate that neuronal activity within the posterior cerebellar cortex plays an important role in the control of alcohol consumption in male mice.

## Significance Statement

The role of the cerebellum in regulating alcohol drinking behavior, independent from motor coordination, is largely unexplored. As cerebellar modulation of nonmotor brain functions is localized primarily to posterior lobules, we chemogenetically perturbed cerebellar lobules to test their involvement in alcohol consumption. Chemogenetic manipulation of posterior Lobules VI–VIII decreased alcohol consumption in male but not female mice and did not alter motor coordination and locomotion. Chemogenetic perturbation of the anterior cerebellum decreased locomotion in both male and female mice but had no effect on motor coordination or alcohol consumption. These findings suggest that in male mice, modulation of the posterior cerebellum may affect neuronal computation within basal ganglia, striatal, and cortical regions known to be relevant in alcohol use disorders.

## Introduction

Because of its extensive connections with functionally diverse brain regions and the great computational power generated by its massive cell number, the cerebellum is a key contributor to CNS processing. Currently, the concept of the cerebellum as a motor control device serves as a model for how the cerebellum also influences autonomic, vestibular, sensorimotor, emotional and cognitive behaviors (Schmahmann, 2021). In the last four decades, the role of the cerebellum, and in particular the posterior cerebellum, in modulating emotion and cognition has gained traction in the neuroscience field (Van Overwalle and Mariën, 2016; Schmahmann, 2019). As these higher order brain functions are critically affected in addiction, research into the potential role of the cerebellum in substance and alcohol use disorders is a growing field of interest, with studies on cocaine, opioid and cannabis use disorders leading the way (Schneider et al., 2001; Moulton et al., 2014; Gil-Miravet et al., 2019; Moreno-Rius, 2019a,b; Miquel et al., 2020; Ranjbar et al., 2021; Guarque-Chabrera et al., 2022).

The mechanisms underlying the cerebellum’s role in addiction-related behaviors has been investigated by manipulating neuronal activity, optical and electrophysiological monitoring, and tracing studies in the rodent cerebellum (Miquel et al., 2016; Wagner et al., 2017; Badura et al., 2018; Xiao et al., 2018; Carta et al., 2019; Gil-Miravet et al., 2019). For example, cerebellar granule cells, the most abundant neuronal subtype in the brain, were found to encode the expectation of reward in mice, thus enriching the contextual information available to postsynaptic Purkinje neurons (PCs), including those associated with drug-induced experiences (Wagner et al., 2017). Interestingly, genetic differences in cerebellar granule cell sensitivity to acute alcohol have been shown to influence alcohol consumption. Thus, alcohol enhances granule cell tonic GABA_A_-mediated currents in strains of low alcohol consuming rodents while suppressing it in high alcohol consuming strains (Kaplan et al., 2016a,b; Erikson et al., 2022). In addition to processing reward-related information, the cerebellum can modulate behavior and neuronal activity in known reward processing brain regions. For instance, reward-driven alternation behavior in mice is truncated by chemogenetic silencing of cerebello-striatal connections (Xiao et al., 2018). Neurons in the deep cerebellar nuclei (DCN) send excitatory monosynaptic projections to the VTA and optogenetic activation of these projections elicited reward related behaviors (Carta et al., 2019). Microstimulation of the DCN elicits short-latency responses in the basolateral amygdala (Heath et al., 1978) and a recent tracing study established the existence of a di-synaptic circuit between cerebellar nuclei and the basolateral amygdala (Jung et al., 2022). Electrical stimulation of the DCN has also been shown to induce dopamine release in the medial prefrontal cortex (mPFC; Mittleman et al., 2008; Rogers et al., 2011).

In the alcohol research field, the cerebellum is known to be particularly sensitive to alcohol, with concentrations as low as 9 mm significantly altering synaptic transmission in the cerebellar cortex (He et al., 2013; Richardson and Rossi, 2017; Zamudio-Bulcock et al., 2018). Moreover, studies in humans indicate that reduced sensitivity to alcohol-induced cerebellum-dependent motor impairment, family history of alcohol use disorder (AUD), and development of AUD are associated (reviewed in (Schuckit et al., 2004). In addition, presentation of alcohol or alcohol related cues induced activation of the cerebellum and other areas in heavy drinking adolescents (Brumback et al., 2015) and adults with AUD who were recently detoxified (Schneider et al., 2001; Olbrich et al., 2006). These responses were correlated with the degree of craving and returned to control levels after three to four weeks of monitored abstinence (Brumback et al., 2015) or cognitive behavioral therapy (Schneider et al., 2001). In light of the accumulating research demonstrating the involvement of posterior cerebellar cortex lobules in drug-related behaviors, we hypothesized that the cerebellum, through its multiple connections to brain circuitry that underlie reward, is important in regulating alcohol consumption. The findings of the present study suggest that activity within posterior cerebellar lobules plays an important role in modulating voluntary alcohol consumption in C57Bl/6J male mice.

## Materials and Methods

### Subjects

Adult seven-week-old C57BL/6J male and female mice were obtained from The Jackson Laboratory. Locomotor behavior (rotarod, open field) was tested before the start of the EtOH drinking paradigms. All experiments using mice were approved by the MUSC Institutional Animal Care and Use Committee and conformed to NIH *Guidelines for the Use of Animals in Biomedical Research*.

### Stereotaxic surgery

Mice were bilaterally injected (250 nl) in the vermal portion of posterior or anterior lobules with an inhibitory designer receptor exclusively activated by designer drugs (DREADDs) vector rAAV8/hSyn-hM4D(Gi)-mCherry (Addgene #50475), AAV8/CaMKIIα-hM4D(Gi)-mCherry (UNC, GTC vector core), or the control vector AAV8/hSyn-mCherry (Addgene #114422). The injection in the anterior cerebellum targeted lobules IV and V [coordinates from bregma (in mm): medial-lateral (ML), ± 0.7, anterior-posterior (AP), −6.48 dorsal-ventral (DV), −2.5] and the injection in the posterior cerebellum encompassed Lobules VI–VIII [coordinates from bregma (in mm): ML ± 0.7, AP, −8.06, DV, −3.9]. Viruses were allowed to express for one month before locomotion or drinking studies were initiated.

### Electrophysiological recordings

Male mice used for electrophysiological validation studies were alcohol naive and stereotaxic injections of the DREADD vector were done three weeks before recordings. Following anesthesia with isoflurane, animals were euthanized and brains were removed and placed in ice-cold oxygenated (95% O_2_, 5% CO_2_) sucrose-containing buffer containing (in mm) 200 sucrose, 1.9 KCl, 1.2 NaH_2_PO_4_, 6 MgCl_2_, 0.5 CaCl_2_, 0.4 ascorbate, 10 glucose, and 25 NaHCO_3_; adjusted to 305–315 mOsm. Sagittal slices containing the cerebellum were cut using a VT1000 vibrating microtome (Leica Biosystems) with a double-walled chamber through which cooled (4°C) solution was circulated. Slices were collected and transferred to a chamber filled with artificial CSF (aCSF) solution containing (in mm): 125 NaCl, 2.5 KCl, 1.4 NaH_2_PO_4_, 2 CaCl_2_, 1.3 MgCl_2_, 10 glucose, 0.4 ascorbic acid, and 25 NaHCO_3_; osmolarity 310–320 mOsm. After at least 1-h incubation at room temperature, whole-cell patch-clamp recordings were acquired using an Axon 700B amplifier (Molecular Devices) controlled by AxographX software (Axograph). The functional effect of activating DREADD receptors in molecular layer interneurons (MLIs) was tested by measuring the firing of adjacent Purkinje neurons given that these cells provide the sole output of the cerebellar cortex and receive inhibitory control from MLIs. DCZ effects on evoked firing were tested as follows. Recording pipettes (resistance of 3–4 MΩ) were filled with internal solution containing (in mm): 120 K-gluconate, 1 EGTA, 10 KCl, 10 HEPES, 2 MgCl_2_, 2Na_2_ATP, and 0.3 Na_3_GTP. Recordings were done in the current-clamp mode and sufficient current was injected to achieve a membrane potential of −70 mV. A depolarizing step of 1 s, 100 pA, was delivered through the patching pipette every 20 s. After a 10-min baseline, 0.2 μm DCZ was bath applied for 10 min. The number of action potentials (APs) elicited by the current injection was quantified and compared between baseline and DCZ application recording periods. The effect of activating inhibitory DREADDs in MLIs was also tested by measuring spontaneous firing of Purkinje neurons using the loose-patch cell-attached configuration. Recording pipettes were filled with aCSF solution and recordings consisted in a 10-min baseline period followed by 10-min bath application of 10 μm clozapine-N-oxide (CNO).

### Drinking

#### Home cage

In a two-bottle choice 24-h intermittent access paradigm, mice were given access to one regular bottle of water for 24 h and on the next day, two bottles containing either 20% EtOH or water were introduced. This pattern of access to EtOH was alternated every 24 h with three weekly drinking sessions starting on Monday, Wednesday, and Friday. Drinking was maintained for at least 30 d. Vehicle (1% DMSO for CNO, or saline for DCZ) was subcutaneously injected 30–45 min before the introduction of bottles for the three consecutive drinking sessions within a given week. Mice were injected with CNO (5 mg/kg) or DCZ (0.2 mg/kg) on testing weeks. Water and EtOH bottles were weighed at 5 h into each session to ensure the measurements were made within the bioavailability period of the DREADD ligands, and EtOH consumption was calculated as grams per kilogram of body weight.

#### Operant

Male mice and female mice were trained to self-administer EtOH on a fixed-ratio one schedule in daily (Monday–Friday) 30-min sessions using a postprandial protocol as previously described (Gioia and Woodward, 2021). Mice were food restricted until 2 h before the start of the daily drinking session and allowed to eat *ad libitum* for 2 h before and 2 h after the drinking session. Water was continuously available until 2 h before drinking sessions. Drinking sessions were conducted in sound-attenuated Med-Associates boxes with the fan and house light turned on at the beginning of each session. During initial training trials, 20 μl of EtOH (15% v/v) was delivered noncontingently to the drinking well every minute using calibrated Med-Associates pumps. Infusions were preceded by auditory (tone) and visual (house light off) cues. In subsequent sessions, an active lever press activated those same auditory and visual cues for 1.5 s while 20 μl of the EtOH (15% v/v) solution were delivered to a drinking well. The active lever had a 1.5-s time out during the presentation of the cue and reward delivery. A second lever in the box (inactive) produced no cues or delivery of EtOH when pressed. Drinking at the alcohol well was monitored using Med-Associates lickometer circuitry and licking microstructure was determined using custom-written functions in MATLAB (The MathWorks) with a drinking bout defined as three or more licks occurring with an interlick interval of <1 s (Renteria et al., 2020).

### Locomotor function

Mice were tested 45 min following injection with either vehicle (1% DMSO) or CNO (5 mg/kg, s.c.) using Med-Associates activity monitors. Mice were habituated to the locomotor boxes for two, 1 h daily sessions. Then, on sessions 3 and 4, mice were administered CNO or vehicle with half the mice receiving CNO on session three and vehicle on session four and vice versa. Total distance traveled over a 1-h test period was measured. Data from male and female mice were not significantly different and therefore were pooled.

### Motor coordination

Mice were trained once a day for 3 d (five trials per session, 2-min rest) in an accelerated rotarod (Ugo Basile) with increasing speed from 5 to 40 rpm in 120 s and a maximum trial time of 300 s. The training sessions were followed by 2 test days where mice were injected with either vehicle or CNO (5 mg/kg, s.c.; counterbalanced across testing sessions) 45 min before being placed on the rotarod. Data from male and female mice were not significantly different and therefore were pooled.

### Tastants drinking

Sucrose (5%) and water drinking were measured in alcohol naive male and female mice expressing either rAAV8/hSyn-hM4D-mCherry or AAV8/hSyn-mCherry using a two-bottle choice 24-h intermittent access paradigm. After one week to establish baseline drinking, mice were injected with either vehicle or CNO (5 mg/kg, s.c.) 45 min before the start of each drinking session for two consecutive weeks. Each mouse received a week of vehicle and a week of CNO injections. Subsequently, quinine (60 μm) drinking was tested using the same drinking and injection paradigm. Data are presented as the average of three drinking sessions in a given week and reported as mean ± SEM.

### Experimental design and statistical analysis

In experiments of cage drinking of alcohol and tastants, injections with the DREADD activators were performed using a within-subject crossover design. Data were analyzed by Prism 9 software (GraphPad Software Inc.) using repeated measures ANOVA followed by *post hoc* comparisons with the *p* value corrected for multiple comparisons using either Tukey’s or Sidak’s *post hoc* tests where indicated and as appropriate. Normalized values were analyzed with one-sample *t* tests using a theoretical mean of 100.

## Results

### DREADD receptor expression and activation in the cerebellar cortex

**Figure 1.**
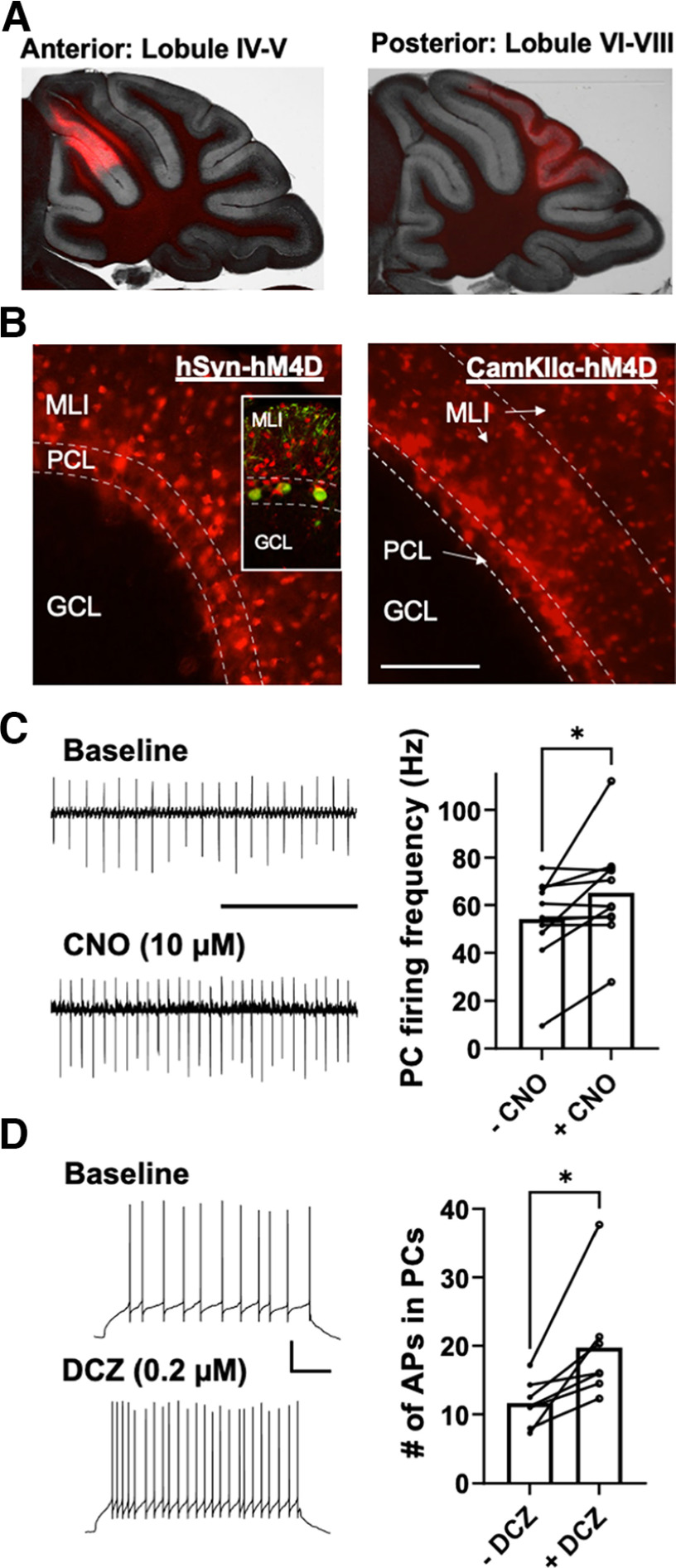
Expression and functional validation of inhibitory DREADDs in anterior and posterior cerebellar cortex lobules. ***A***, Images show representative examples of expression of rAAV8/hSyn-hM4D-mCherry in anterior (left) and posterior (right) lobules of the cerebellum. ***B***, Images show hM4D-mCherry expression in molecular layer interneurons (MLIs) in the cerebellar cortex in mice infused with AAV constructs under the control of the hSyn (left) or CaMKIIα (right) promoters. Inset on left panel shows calbindin-labeled Purkinje neurons (green) in a section expressing hM4D-mCherry in MLIs. ***C***, Sample traces show effect of the DREADD agonist CNO (10 μm) on spontaneous firing of a Purkinje neuron within a region expressing hSyn-hM4D in MLIs; scale: 0.2 s. Summary plot shows CNO-induced increase in PC firing (*p <* *0.05; *N* = 11). ***D***, Sample traces show effect of the DREADD agonist DCZ (0.2 μm) on current-evoked firing of a Purkinje neuron adjacent to hSyn-hM4D-expressing MLIs; scale: 20 mV/100 ms. Summary plot shows DCZ-induced increase in PC firing (*p <* *0.05; *N* = 7). Data are mean ± SEM.

To test the role of the cerebellum in voluntary alcohol consumption, male and female C57BL/6J mice received stereotaxic injections of an inhibitory DREADD (rAAV8/hSyn-hM4D(Gi)-mCherry or AAV8/CaMKIIα-hM4D(Gi)-mCherry) or a control vector AAV8/hSyn-mCherry (hSyn-Cherry), in defined vermal regions of anterior or posterior lobules. Injections in the anterior lobules were placed mid-region between Lobules IV and V while those in posterior lobules were located in Lobules VI, VII, and VIII (Fig. 1*A*). Expression of both CaMKIIα-hM4D and hSyn-hM4D vectors was restricted to molecular layer interneurons (Fig. 1*B*). For the hSyn-hM4D vector, this cell-type selectivity is consistent with that reported for rAAV vectors containing the human synapsin promoter (Kuhn et al., 2012; Badura et al., 2018). However, an AAV1 vector with the CaMKIIα promoter was reported to result in expression in MLIs, but also in Purkinje neurons in adult mice (Kim et al., 2015). This inconsistency could be because of the difference in serotype employed by the Kim and colleagues study and the present study as this is known to affect the cell-type specificity of expression (Hammond et al., 2017). To assess changes in PC firing on inhibitory DREADD receptor activation, whole-cell and loose-cell-attached patch-clamp recordings of PCs were performed in acute slice preparations under baseline conditions and during bath application of CNO or DCZ. In recordings using CNO, the last 3 min of a 10-min baseline recording was averaged and compared with the firing frequency in the last 3 min of a 10-min CNO (10 μm) bath application. CNO induced a significant increase in the firing frequency of PCs by (Baseline, 54.17 ± 5.38 Hz; CNO, 65.21 ± 6.32 Hz; paired *t* test, *p* = 0.038, *n* = 11 cells, 4 mice; Fig. 1*C*). In current-step recordings using the DREADD receptor agonist DCZ, the number of action potentials (APs) elicited by a 1 s, 100-pA current injection was quantified in the 3 min before DCZ bath application and in the last 3 min in DCZ. As shown in Figure 1*C*, the average number of APs in the presence of DCZ was significantly greater than the number of APs during baseline (DCZ, 19.7 ± 3.2; Baseline, 11.64 ± 1.3; paired *t* test, *p* = 0.02, *n* = 7 cells, 4 mice; Fig. 1*D*).

### Chemogenetic inhibition of posterior cerebellar lobules decreases alcohol consumption in male, but not female, mice

**Figure 2.**
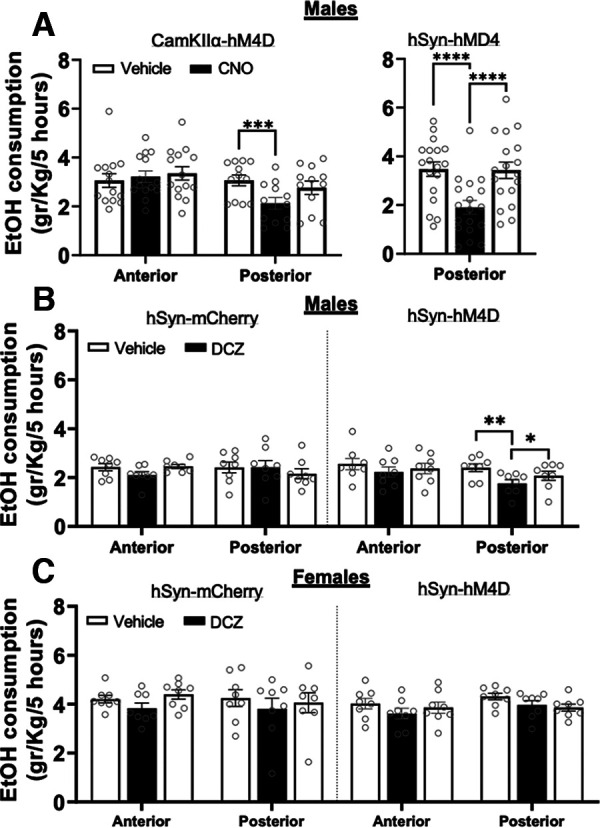
Chemogenetic inhibition of cerebellar Lobules VI–VIII, but not IV–V decreases voluntary EtOH consumption in male mice. ***A***, Weekly average EtOH consumption following administration of vehicle or CNO (5 mg/kg, s.c.) in male mice expressing CaMKIIα-hM4D (left panel) in anterior (*N* = 14) or posterior cerebellar cortex lobules (*N* = 12, vehicle baseline vs CNO, ***p =* *0.0003, Tukey’s multiple comparisons); or hSyn-hM4D (right panel) in posterior lobules (*N* = 18, ****p <* *0.0001, Sidak’s multiple comparisons). ***B***, Weekly average EtOH consumption after vehicle or DCZ injections (0.2 mg/kg, s.c.) in mice expressing the control viral vector hSyn-mCherry (left panel) or hSyn-hM4D (right panel) in anterior or posterior lobules. DCZ decreased the amount of EtOH consumed in mice expressing the inhibitory DREADD receptors in posterior but not anterior lobules amounts when compared with vehicle injections the week prior and the week following DCZ treatment (**p <* *0.0068, *p <* *0.03, Tukey’s multiple comparisons). ***C***, Summary of the average EtOH consumption after vehicle and DCZ injections in female mice expressing hSyn-mCherry (left, *N* = 8) or hSyn-hM4D (right, *N* = 8), in anterior or posterior cerebellar cortex lobules. Data represent the average of the three drinking sessions in a given week and are expressed as the mean ± SEM.

After verifying that activation of inhibitory DREADD receptors expressed in MLIs significantly increases PC firing, we tested whether CNO and DCZ also affect voluntary ethanol consumption. Male mice expressing an inhibitory DREADD in the posterior but not anterior cerebellum showed a significant decrease in alcohol consumption when the DREADD receptor was activated. Figure 2*A*, left panel, shows the weekly average ethanol consumption in mice expressing CaMKIIα -hM4D in anterior (IV–V) lobules or posterior (VI–VIII) lobules following an injection of vehicle or CNO (5 mg/kg), 45 min before the start of the drinking session. There was a significant interaction between the amount of ethanol consumed and the cerebellar region and injection (veh/CNO; *F*_(2,48)_ = 4.85, *p *=* *0.012, two-way repeated measures (RM) ANOVA). Pairwise comparisons showed a statistically significant difference in alcohol consumption between vehicle and CNO injection in mice expressing CaMKIIα -hM4D in posterior cerebellar lobules (*p = *0.0003, 95% confidence interval (CI) of diff. 0.51–1.37, *n* = 12, Tukey’s multiple comparisons test; Fig. 2*A*, left panel). In a separate cohort of mice expressing hSyn-hM4D in the posterior cerebellum, CNO induced a similar decrease in ethanol consumption (*F*_(1.97,33.4)_ = 29.24, *p* < 0.0001, one-way RM ANOVA; Fig. 2*A*, right panel). Pairwise comparisons showed that, after CNO injection, mice consumed significantly less ethanol as compared with consumption following vehicle the week before (*p *<* *0.0001, 95% CI of diff. 0.91–2.23, *n* = 18, Bonferroni’s multiple comparisons test), or the week after the CNO injection (*p *<* *0.0001, 95% CI of diff. −2.1 to −0.94, *n* = 18, Bonferroni's multiple comparisons test).

In a third cohort of male and female mice, we used hSyn-hM4D and a control hSyn-mCherry viral vector to further investigate the regional and sex-dependent effects of cerebellar DREADD activation on ethanol drinking. In this experiment, we activated DREADDs using DCZ. Male and female mice expressing the control hSyn-mCherry or hSyn-hM4D construct in anterior or posterior cerebellar lobules received saline or DCZ (0.2 mg/kg, s.c.) injections 30 min before each drinking session. In male mice, there was a significant interaction between cerebellar region, viral vector and injection treatment (*F*_(2,56)_ = 5.43, *p* = 0.007, three-way RM ANOVA). Pairwise comparisons showed reduced ethanol consumption after DCZ injection in male mice expressing hSyn-hM4D in posterior but not anterior cerebellar lobules as compared with vehicle injection during baseline the week before (*p *=* *0.007, 95% CI of diff. 0.2–1, *n* = 8, Tukey’s multiple comparisons test), or after DCZ treatment (*p *=* *0.03, 95% CI of diff. −0.6–0.03, *n* = 8, Tukey’s multiple comparisons test; Fig. 2*B*). In contrast, DCZ had no effect on ethanol drinking in female mice (*F*_(2,56)_ = 0.04, *p* = 0.96, three-way RM ANOVA; Fig. 2*C*).

For all male mice expressing hM4Ds or the control mCherry vector in posterior cerebellar lobules, ethanol consumption was normalized to each animal’s baseline drinking. Overall, DCZ/CNO treatment significantly reduced ethanol consumption in mice expressing hM4D (normalized DCZ/CNO: 62 ± 3.6% of baseline, one-sample *t* test *p* < 0.0001, *n* = 38), but not in those expressing the control vector (normalized DCZ: 99.56 ± 5.37% of baseline, one-sample *t* test *p* = 0.94, *n* = 8; data not shown).

### Inhibitory DREADD receptor activation in anterior and posterior lobules does not significantly affect locomotor activity

**Figure 3.**
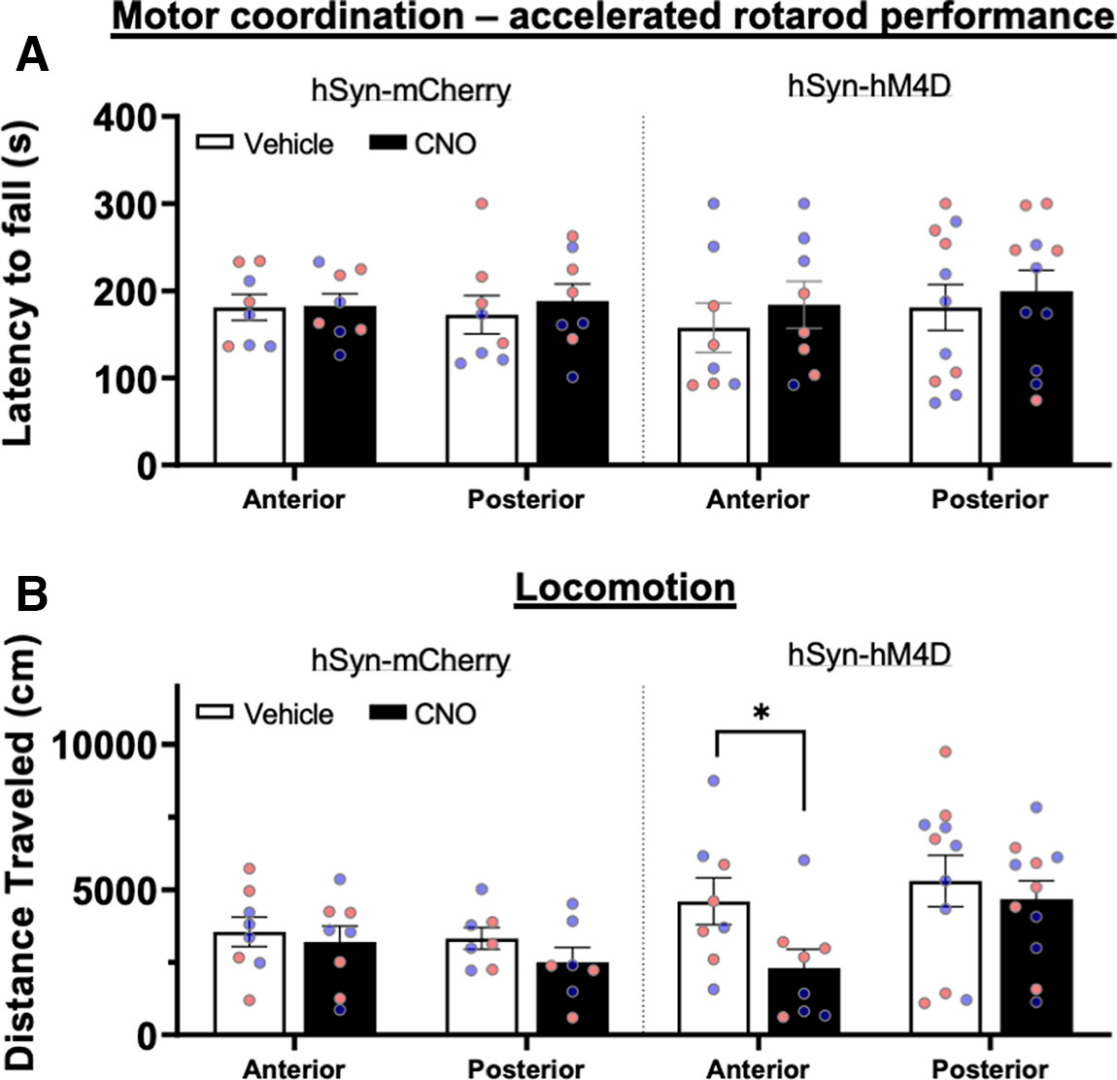
Effect of chemogenetic perturbation of the cerebellar cortex on locomotor activity in male and female mice. ***A***, Male (blue symbols) and female (pink symbols) mice expressing rAAV8/hSyn-hM4D-mCherry or the control virus AAV8/hSyn-mCherry in anterior and posterior cerebellar cortex lobules showed no changes in the latency to fall in the accelerated rotarod task following injection of CNO (5 mg/kg, s.c.). ***B***, Open field activity as measured by the total distance traveled in locomotor boxes was significantly reduced by CNO (5 mg/kg, s.c.) in mice expressing inhibitory DREADD receptors in the anterior (*p =* *0.013) but not the posterior cerebellar cortex. Data are mean ± SEM (*N* = 7–11).

Given the cerebellum’s central role in movement and balance, it was important to test whether chemogenetic inhibition of MLIs produced aberrant locomotion that could interfere with drinking behavior. For this control study, we used CNO to activate the inhibitory DREADD receptor given that CNO has been reported to be back metabolized to clozapine that can interact with a variety of neurotransmitter receptors including those activated by serotonin and dopamine (Gomez et al., 2017); such off target effects could alter behavior independently of DREADD receptor activation. We first tested whether DREADD activation affected motor coordination in an accelerated rotarod task in alcohol naive, male and female mice expressing hSyn-hM4D-mCherry or the control virus hSyn-mCherry in anterior (IV–V) or posterior (VI–VIII) cerebellar lobules. For the combined male and female data (Fig. 3*A*), there was no interaction between cerebellar region, viral vector and injection treatment on the latency to fall (*F*_(1,31)_ = 0.49, *p* = 0.49, three-way RM ANOVA; Fig. 3*A*). To test for sex differences, we separately analyzed data for the anterior and posterior lobules. The latency to fall from the rotorod did not differ between males and females and there were no significant interactions between viral construct, injection treatment and sex (anterior: *F*_(1,12)_ = 0.013, *p* = 0.91, three-way RM ANOVA; posterior: *F*_(1,15)_ = 1.30, *p* = 0.27, three-way RM ANOVA).

General locomotor activity was tested with Med-Associates activity monitors following two habituation sessions in the chamber. When the combined data from male and female mice were analyzed, we found no significant interaction between cerebellar region, viral vector and treatment (*F*_(1,30)_ = 2.91, *p* = 0.1, three-way ANOVA) on total distance traveled. However, there was a simple main effect of treatment on distance traveled (*F*_(1,30)_ = 10.63, *p* = 0.0028, three-way RM ANOVA; Fig. 3*B*). Pairwise comparisons showed a significant reduction in distance traveled after CNO injection when compared with that of vehicle injection in mice expressing hSyn-hM4D in anterior cerebellar lobules (*p *=* *0.014, 95% CI of diff. 320.2–4293, *n* = 8, Sidak’s multiple comparisons test; Fig. 3*B*). To test for sex differences, we separately analyzed data for the anterior and posterior lobules. In the anterior cerebellum there was no significant interaction between treatment, viral vector and sex (*F*_(1,12)_ = 0.44, *p* = 0.52). However, there was a simple main effect of CNO on distance traveled (*F*_(1,12)_ = 5.7, *p* = 0.034, three-way RM ANOVA), but pairwise comparisons revealed no significant effects between groups. In the posterior cerebellum there was also no significant interaction between treatment, viral vector and sex (*F*_(1,14)_ = 0.37, *p* = 0.55) on distance traveled and no main effect of CNO treatment (*F*_(1,14)_ = 3.88, *p* = 0.069, three-way RM ANOVA), sex (*F*_(1,14)_ = 0.17, *p* = 0.68, three-way RM ANOVA) or viral construct (*F*_(1,14)_ = 4.29, *p* = 0.057, three-way RM ANOVA).

### Chemogenetic perturbation in the posterior cerebellar cortex does not alter sucrose or quinine voluntary consumption in male and female mice

**Figure 4.**
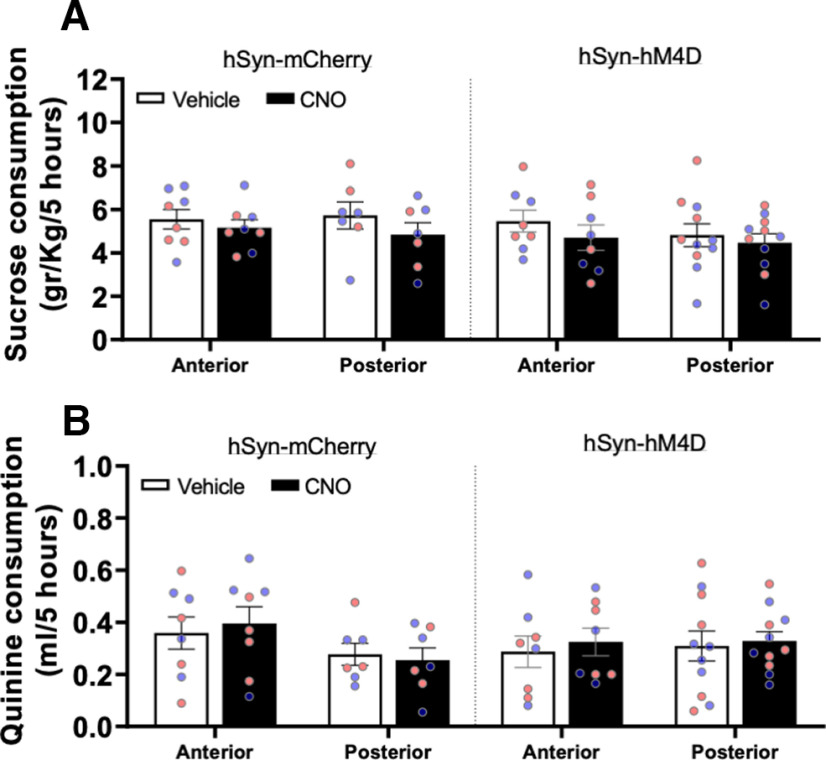
Chemogenetic perturbation of the posterior cerebellar cortex does not alter sucrose or quinine voluntary consumption in male or female mice. Graphs show average consumption of 5% sucrose (***A***) or 60 μm quinine (***B***) after vehicle or CNO injection in male (blue symbols) and female (pink data symbols) mice expressing rAAV8/hSyn-hM4D-mCherry (right panels, ***A***, ***B***) or AAV8/hSyn-mCherry (left panels, ***A***, ***B***) in anterior and posterior cerebellar cortex lobules. Data are the average of the three drinking sessions in a given week and represent the mean ± SEM (*N* = 7–11).

In a separate cohort of male and female alcohol naive mice, we assessed whether chemogenetic perturbation of anterior or posterior cerebellar cortex activity would affect drinking of appetitive (5% sucrose) or aversive (60 μm quinine) solutions. For these studies, we used the same homecage intermittent two-bottle choice drinking paradigm described previously and drinking amounts were recorded 5 h after the start of each session. Sucrose/quinine drinking started one week before vehicle/CNO injections. When the combined male and female data were analyzed, there was no significant interaction between cerebellar region, viral vector and injection treatment on sucrose drinking (*F*_(1,30)_ = 1.4, *p* = 0.24, three-way RM ANOVA; Fig. 4*A*, *N* = 7–11). There was a simple main effect of CNO on sucrose drinking (*F*_(1,30)_ = 9.7, *p* = 0.004, three-way RM ANOVA), but follow-up pairwise comparisons showed no significant differences between any of the groups. As in the locomotion studies, we separately analyzed data for the anterior and posterior lobules to assess sex differences. In mice expressing virus in the anterior cerebellum there was no significant interaction between the amount of sucrose consumed and sex, injection treatment and viral vector (*F*_(1,12)_ = 0.0095, *p *=* *0.92, three-way RM ANOVA) and a similar finding was found for the posterior cerebellum (*F*_(1,14)_ = 3.2, *p *=* *0.095, three-way RM ANOVA). For quinine drinking, there was no significant interaction between viral vector, injection treatment and cerebellar region (*F*_(1,30)_ = 0.053, *p* = 0.84, three-way RM ANOVA, *N* = 7–11; Fig. 4*B*) in the combined male and female drinking data. When tested for sex differences, there was no significant interaction between and sex, injection treatment and viral vector for the amount of quinine consumed in either the anterior (*F*_(1,12)_ = 0.42, *p *=* *0.53, three-way RM ANOVA); or posterior cerebellum (*F*_(1,14)_ = 0.024, *p *=* *0.88, three-way RM ANOVA).

### Activation of inhibitory DREADDs on MLIs in the posterior cerebellum decreases operant self-administration of EtOH in male but not female mice

To test whether chemogenetic alteration of the posterior cerebellum affected responding for alcohol, drinking was conducted using an operant ethanol self-administration paradigm in male and female mice expressing either the mCherry control virus or the hSyn-hM4D vector. After eight weeks of baseline operant sessions, mice received saline or DCZ (0.2 mg/kg, s.c.) injections 30 min before each drinking session. Mice received saline injections the week prior and the week after DREADD receptor activation sessions. Drinking microstructure was analyzed using the following parameters: number of licks, number of drinking bouts (defined as three or more licks with <1 s between licks), latency from active lever presses to the start of licking bouts and lick rate during a bout in the weekly average of the 30-min operant drinking sessions.

**Figure 5.**
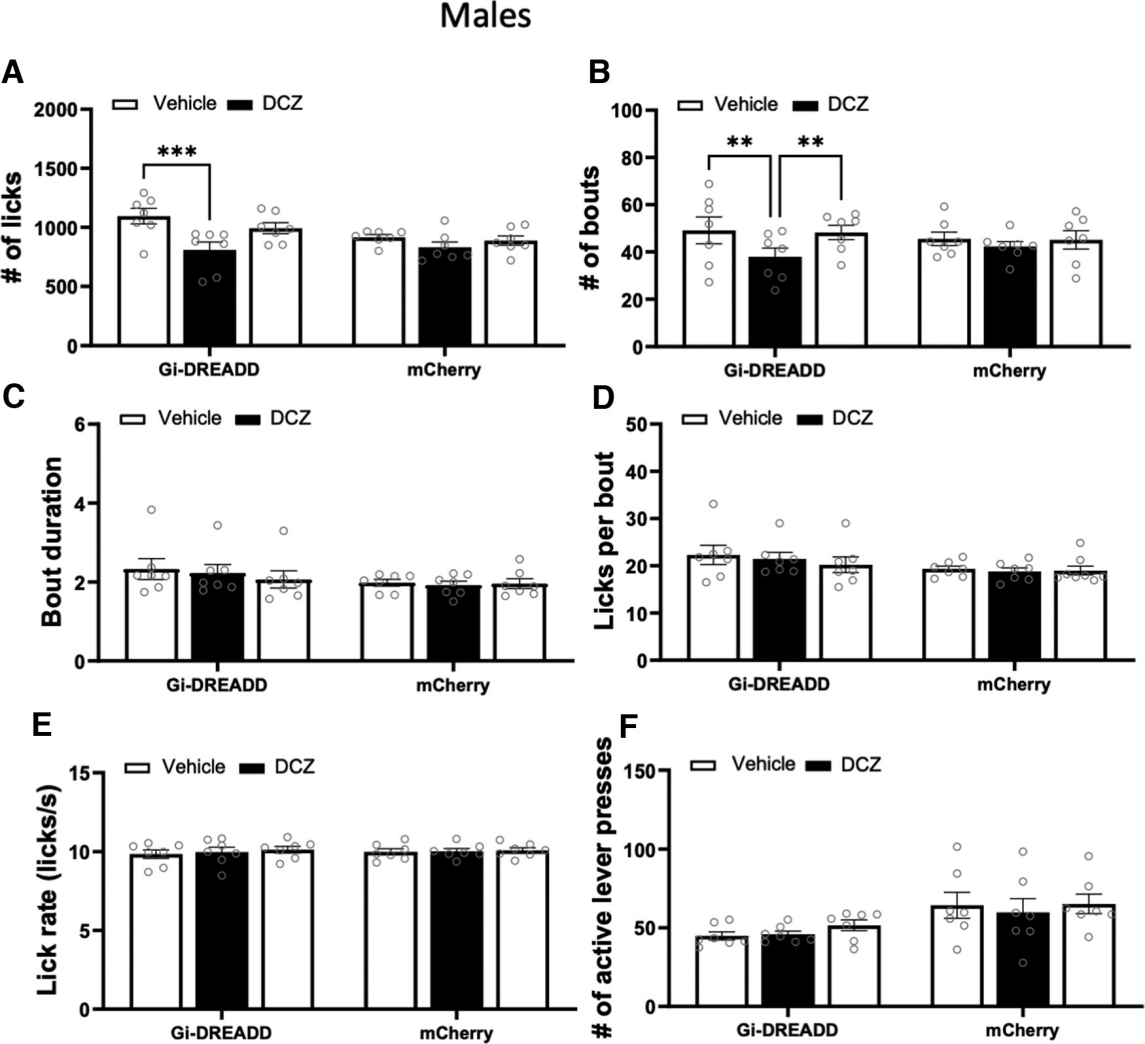
Chemogenetic inhibition of cerebellar Lobules VI–VIII in male mice reduces ethanol drinking during operant self-administration sessions. Graphs show the microstructure of ethanol drinking after vehicle and DCZ injections (0.2 mg/kg, s.c.) in male mice expressing rAAV8/hSyn-hM4D-mCherry or AAV8/hSyn-mCherry in posterior cerebellar cortex lobules. ***A***, Number of licks per session (Vehicle Baseline vs DCZ: ***p =* *0.0009, Tukey’s multiple comparisons). ***B***, Number of drinking bouts per session (Vehicle Baseline vs DCZ: **p =* *0.0068 and DCZ vs Vehicle: **p =* *0.0030, Tukey’s multiple comparisons). ***C***, Bout duration in seconds. ***D***, Number of licks per bout. ***E***, Rate of licking per session. ***F***, Number of active lever presses. Data represent the average of daily drinking sessions in a given week and are expressed as the mean ± SEM (*N* = 7).

For male mice, a two-way ANOVA analysis revealed a significant interaction between the viral construct and treatment on the number of licks (veh/DCZ; *F*_(2,24)_ = 3.76, *p *=* *0.038, two-way RM ANOVA). Pairwise comparisons showed a statistically significant difference in the number of licks between baseline vehicle and DCZ injection in mice expressing hSyn-hM4D (*p = *0.0009, 95% CI of diff. 162.4–407.4, *n* = 7, Tukey’s multiple comparisons test; Fig. 5*A*) but not in those expressing the mCherry control virus (*p* = 0.25). For the drinking bouts data, there was a simple main effect of treatment (veh/DCZ; *F*_(1.76,21.17)_ = 5.48, *p *=* *0.015, two-way RM ANOVA) and pairwise comparisons revealed a statistically significant difference in bout number between baseline vehicle and DCZ treatment in mice expressing hSyn-hM4D (*p = *0.007, 95% CI of diff. 4.12–18.28, *n* = 7, Tukey’s multiple comparisons test; Fig. 5*B*) and between DCZ injection and vehicle treatment the following week (*p = *0.003, 95% CI of diff. −15.81 to −4.76, *n* = 7, Tukey’s multiple comparisons test; Fig. 5*B*). There was no difference in bout number following DCZ treatment in the mCherry control mice. In regards to bout duration there was no interaction between treatment and viral vector (*F*_(2,24)_ = 1.82, *p *=* *0.18, two-way RM ANOVA; Fig. 5*C*). Similarly, the number of licks per bout was not altered by treatment and viral vector (*F*_(2,24)_ = 1.4, *p *=* *0.27, two-way RM ANOVA; Fig. 5*D*). There was also no interaction between treatment and viral vector on the rate of licking during ethanol self-administration (*F*_(2,24)_ = 0.85, *p *=* *0.44, two-way RM ANOVA; Fig. 5*E*). Analysis of the lever press data indicated no interaction between treatment and viral vector on the number of active or inactive lever presses (active lever presses: *F*_(2,24)_ = 0.86, *p *=* *0.44, two-way RM ANOVA; Fig. 5*F*; inactive lever presses *F*_(2,24)_ = 2.15, *p *=* *0.14, two-way RM ANOVA; data not shown).

Given the decrease in the average number of licks and drinking bouts in inhibitory DREADD expressing male mice on DCZ injections, we calculated the amount of time elapsed between active lever presses and the start of drinking bouts. Analysis of lever press to drinking latency data revealed a nearly significant interaction between treatment and viral vector (*F*_(2,24)_ = 3.34, *p *=* *0.053, two-way RM ANOVA; data not shown) suggesting that, after DCZ injections, DREADD expressing male mice took longer to initiate a drinking bout following an active lever press. However, pairwise comparisons found no significant differences between groups.

**Figure 6.**
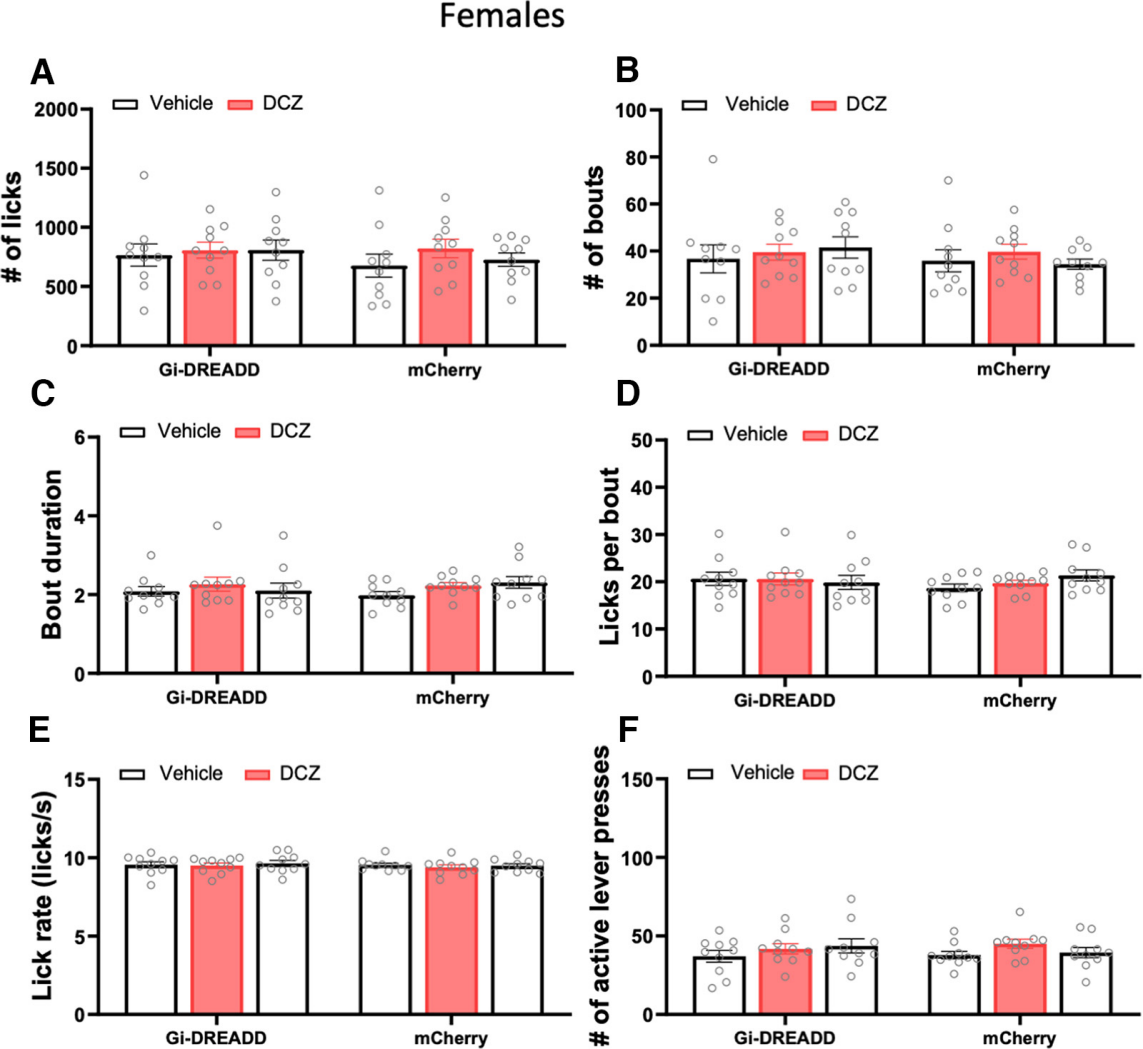
Chemogenetic inhibition of cerebellar lobules in female mice VI–VIII does not affect ethanol drinking during operant self-administration sessions. Graphs show the microstructure of ethanol drinking after vehicle and DCZ injections (0.2 mg/kg, s.c.) in female mice expressing rAAV8/hSyn-hM4D-mCherry or AAV8/hSyn-mCherry in posterior cerebellar cortex lobules. ***A***, Number of licks per session. ***B***, Number of drinking bouts per session. ***C***, Bout duration in seconds. ***D***, Number of licks per bout. ***E***, Rate of licking per session. ***F***, Number of active lever presses. Data represent the average of daily drinking sessions in a given week and are expressed as the mean ± SEM (*N* = 10).

For female mice, there was no interaction between treatment and viral vector on the number of licks made during the operant ethanol self-administration sessions (*F*_(2,36)_ = 0.31, *p *=* *0.73, two-way RM ANOVA; Fig. 6*A*). Similarly, there was no interaction between treatment and viral vectors on the number of bouts (*F*_(2,36)_ = 0.78, *p *=* *0.46, two-way RM ANOVA; Fig. 6*B*), bout duration (*F*_(2,36)_ = 2, *p *=* *0.15, two-way RM ANOVA; Fig. 6*C*), licks per bout (*F*_(2,36)_ = 1.95, *p *=* *0.16, two-way RM ANOVA; Fig. 6*D*), lick rate (*F*_(2,36)_ = 0.57, *p *=* *0.57, two-way RM ANOVA; Fig. 6*E*), number of active lever presses (*F*_(2,36)_ = 1.5, *p *=* *0.23, two-way RM ANOVA; Fig. 6*F*) and number of inactive lever presses (*F*_(2,36)_ = 1.51, *p *=* *0.23, two-way RM ANOVA; data not shown).

## Discussion

In this study, an inhibitory DREADD was expressed in molecular layer interneurons in discrete anterior or posterior lobules of male and female C57B/l6J mice to examine whether modulating cerebellar activity affects EtOH drinking. In the posterior, but not anterior cerebellar cortex, activation of an inhibitory DREADD significantly reduced alcohol consumption during either home-cage drinking or operant self-administration. This effect was only observed in male mice as drinking by female mice was unaffected by the DREADD ligand. Similarly, mice expressing the control mCherry vector in either anterior or posterior cerebellar lobules did not show any significant changes in alcohol consumption following CNO/DCZ injection. These results suggest that activity within cerebellar cortex Lobules VI–VIII is important for modulating the amount of alcohol consumption. Importantly, this effect is unlikely to be because of changes in locomotor performance as: (1) activation of inhibitory DREADDs in the posterior cerebellum did not affect rotarod locomotor performance or locomotor ambulatory activity and; (2) activation of inhibitory DREADDs significantly reduced the number of licks and the number of drinking bouts per session, without altering the frequency of licking, the number of licks per bout or the bout duration. These findings suggest that changes in motor coordination, ambulatory locomotion or oromotor performance are likely not responsible for the decrease in voluntary alcohol consumption after DREADD activation. Moreover, the lack of effect of DREADDs on the consumption of aversive or appetitive tastants suggest that the effect on ethanol consumption is not because of a change in the gustatory properties of alcohol. Additionally, although male and female mice expressing the inhibitory DREADD in the anterior cerebellum showed a decrease in open field ambulatory behavior, this was not associated with reduction in EtOH consumption.

Posterior cerebellar cortex Lobules VI–VIII have been linked to AUD relevant behaviors and brain regions. In the present study, the inhibitory hM4D(Gi)-DREADD receptor was selectively expressed in inhibitory MLIs resulting in enhanced firing of PCs during exposure to the DREADD agonists CNO or DCZ. It has been previously shown that chemogenetic inhibition of MLIs in posterior lobules enhances the output of inhibitory Purkinje neurons leading to alterations in reversal learning (Lobule VI), persistent behavior and novelty seeking (Lobule VII), and social preference (Crus I/II; Badura et al., 2018). Interestingly, posterior cerebellar connectivity with striatal and cortical regions has been described and likely contributes to the effects reported by Badura et al., For example, electrical stimulation in the prelimbic subdivision of the mPFC elicits field potentials in the vermal portion of posterior cerebellar Lobule VII (Watson et al., 2009) and anterograde transynaptic labeling of Lobules VI and VII results in high density labeling of neurons within prelimbic, infralimbic and orbital frontal cortices (Badura et al., 2018; Pisano et al., 2021). Furthermore, optogenetic inhibition of PCs in Lobule VI results in enhanced neuronal activation, as measured by c-Fos expression, in various regions of the neocortex, nucleus accumbens, and thalamus (Pisano et al., 2021). Similarly, ablating cerebellar cortex output with an excitotoxic lesion of posterior Lobule VIII results in enhanced c-Fos expression in the lateral nucleus of the deep cerebellar nuclei, the infralimbic and prelimbic prefrontal cortices, the nucleus accumbens, and the dorsomedial and dorsolateral striatum (Gil-Miravet et al., 2021). Interestingly, such a lesion in Lobule VIII increased the probability of cocaine-induced conditioned preference acquisition (Carbo-Gas et al., 2017), suggesting a role for the posterior cerebellum in drug reward.

In addition to the traditional cerebello-thalamo-cortical pathways, noncanonical connections between the cerebellum, the basal ganglia, and the frontal cortex have been recently elucidated (reviewed by Washburn et al., 2022). The DCN connects to the dorsal striatum via two separate ways; one contributes to motor coordination and is comprised of fast glutamatergic signals conveyed via the central lateral nucleus and the parafascicular nucleus of the thalamus. A second pathway is via the substantia nigra pars compacta, that appears to convey information regarding reward value (Washburn et al., 2022). In addition, the DCN connects to the nucleus accumbens via the VTA; activation of this pathway drives dopamine release (Holloway et al., 2019) and elicits inhibitory as well as excitatory responses in the nucleus accumbens core and shell regions (D’Ambra et al., 2021). Thus, in the present study, DREADD-induced enhancement of PC inhibitory drive onto glutamatergic DCN neurons could reduce excitatory output to downstream regions thereby altering synaptic communication within AUD-relevant circuitry. Future studies can test this hypothesis by analyzing changes in neuronal activation in regions downstream of the posterior cerebellum on DREADD receptor activation and by targeting Lobules VI, VII, and VIII separately to dissect out the precise neuronal network underlying the effects reported in the present study.

Various rodent models of alcohol exposure and consumption have shown sex-specific differences in the amounts of alcohol consumed, aversion resistance, alcohol seeking behaviors, magnitude and direction of alcohol-induced neuronal activity changes, and brain neuronal network adaptations (Fulenwider et al., 2019; Xie et al., 2019; Nimitvilai et al., 2020; Alper et al., 2022; Leyrer-Jackson et al., 2022; Foo et al., 2023; Nimitvilai-Roberts et al., 2023; Sneddon et al., 2023). These differences are likely because of distinct neurophysiology and brain connectivity in males versus females. In humans, resting-state functional magnetic resonance imaging showed marked sex differences in resting state cerebellar connectivity to various cortical regions, with females having a lower correlation coefficient between cerebellar clusters and every other voxel in the brain (Smith et al., 2019). It is then likely that in female mice, the neuronal circuitry underlying motivation for alcohol consumption is not significantly modulated by activity in the posterior cerebellar cortex. It is interesting to note that in nondependent mice, females display greater compulsive alcohol seeking than males (Xie et al., 2019), suggesting inherent sex differences in the neurophysiological mechanisms underlying alcohol-related reward and goal directed behaviors. Based on these findings, it is perhaps not surprising that female mice would be resistant to neuronal activity perturbations that alter alcohol consumption in male mice. Follow-up studies can investigate whether there are sex differences in neuronal activation patterns following chemogenetic perturbation of the posterior cerebellar cortex.

Taken together, the results presented herein contribute to the growing pool of studies supporting an important role of synaptic integration and neuronal computation within the posterior cerebellar cortex in behaviors relevant to alcohol and substance use disorders. They also, for the first time, demonstrate that posterior cerebellar lobules modulate alcohol consumption in mice and may potentially contribute to loss of control over drinking that is often observed in individuals with alcohol use disorder.

## Synthesis

Reviewing Editor: Tod E Kippin, University of California Berkeley Decisions are customarily a result of the Reviewing Editor and the peer reviewers coming together and discussing their recommendations until a consensus is reached. When revisions are invited, a fact-based synthesis statement explaining their decision and outlining what is needed to prepare a revision will be listed below. The following reviewer(s) agreed to reveal their identity: Mark Brodie. Note: If this manuscript was transferred from JNeurosci and a decision was made to accept the manuscript without peer review, a brief statement to this effect will instead be what is listed below.

I am happy to inform you that both reviewers and myself no longer have concerns with this manuscript. As such, I am recommending publication.
